# Fiber fractions, multielemental and isotopic composition of a tropical C_4_ grass grown under elevated atmospheric carbon dioxide

**DOI:** 10.7717/peerj.5932

**Published:** 2019-02-19

**Authors:** Adibe L. Abdalla Filho, Geovani T. Costa Junior, Paulo M.T. Lima, Amin Soltangheisi, Adibe L. Abdalla, Raquel Ghini, Marisa C. Piccolo

**Affiliations:** 1 Universidade de São Paulo, Centro de Energia Nuclear na Agricultura—Laboratório de Ciclagem de Nutrientes, Piracicaba, São Paulo, Brazil; 2 Universidade de São Paulo, Centro de Energia Nuclear na Agricultura—Laboratório de Instrumentação Nuclear, Piracicaba, São Paulo, Brazil; 3 Universidade de São Paulo, Centro de Energia Nuclear na Agricultura—Laboratório de Nutrição Animal, Piracicaba, São Paulo, Brazil; 4 Universidade de São Paulo, Centro de Energia Nuclear na Agricultura—Laboratório de Ecologia Isotópica, Piracicaba, São Paulo, Brazil; 5 Embrapa Meio Ambiente, Jaguariúna, Sao Paulo, Brazil

**Keywords:** *Brachiaria decumbens*, Climate change, FACE, Livestock, Calcium

## Abstract

**Background:**

Brazil has the largest commercial herd of ruminants with approximately 211 million head, representing 15% of world’s beef production, in an area of 170 million hectares of grasslands, mostly cultivated with *Brachiaria* spp. Although nutrient reduction due to increased atmospheric carbon dioxide (CO_2_) concentration has already been verified in important crops, studies evaluating its effects on fiber fractions and elemental composition of this grass genus are still scarce. Therefore, a better understanding of the effects of elevated CO_2_ on forage quality can elucidate the interaction between forage and livestock production and possible adaptations for a climate change scenario. The objective of this study was to evaluate the effects of contrasting atmospheric CO_2_ concentrations on biomass production, morphological characteristics, fiber fractions, and elemental composition of *Brachiaria decumbens* (cv. Basilisk).

**Methods:**

A total of 12 octagonal rings with 10 m diameter were distributed in a seven-ha coffee plantation and inside each of them, two plots of 0.25 m^2^ were seeded with *B. decumbens* (cv. Basilisk) in a free air carbon dioxide enrichment facility. Six rings were kept under natural conditions (≈390 μmol mol^−1^ CO_2_; Control) and other six under pure CO_2_ flux to achieve a higher concentration (≈550 μmol mol^−1^ CO_2_; Elevated CO_2_). After 30 months under contrasting atmospheric CO_2_ concentration, grass samples were collected, and then splitted into two portions: in the first, whole forage was kept intact and in the second portion, the leaf, true stem, inflorescence and senescence fractions were manually separated to determine their proportions (%). All samples were then analyzed to determine the fiber fractions (NDF, hemicellulose, ADF, cellulose, and Lignin), carbon (C), nitrogen (N), potassium (K), calcium (Ca), sulfur (S), phosphorus (P), iron (Fe), and manganese (Mn) contents and N isotopic composition.

**Results:**

Elevated atmospheric CO_2_ concentration did not influence biomass productivity, average height, leaf, stem, senescence and inflorescence proportions, and fiber fractions (*p* > 0.05). Calcium content of the leaf and senescence portion of *B. decumbens* were reduced under elevated atmospheric CO_2_ (*p* < 0.05). Despite no effect on total C and N (*p* > 0.05), lower C:N ratio was observed in the whole forage grown under elevated CO_2_ (*p* < 0.05). The isotopic composition was also affected by elevated CO_2_, with higher values of δ^15^N in the leaf and stem portions of *B. decumbens* (*p* < 0.05).

**Discussion:**

Productivity and fiber fractions of *B. decumbens* were not influenced by CO_2_ enrichment. However, elevated CO_2_ resulted in decreased forage Ca content which could affect livestock production under a climate change scenario.

## Introduction

Fossil fuel combustion, land use changes, and the expansion of population and industry have significantly contributed to the global carbon dioxide (CO_2_) rise, from the preindustrial level of 280 ppm to the current level of 400 ppm (International Panel on Climate Change (IPCC)—Climate Change, 2014; Broberg, Högy & Pleijel, 2017), and this increase is expected to continue. According to the representative concentration pathways (RCPs) of International Panel on Climate Change (2014), the atmospheric CO_2_ concentration is estimated to reach the range of 420 ppm (RCP2.6) to 1,300 ppm (RCP8.5) in the next decades. Such increases in CO_2_ concentration is expected to have cascading effects on numerous aspects of plant biochemistry, since plant productivity is strongly tied to atmospheric CO_2_ through photosynthesis (Dietterich et al., 2015).

Experimental studies simulating future scenarios predict that C_4_ species are less responsive to elevated CO_2_ conditions in comparison with C_3_ species due to the differences in their photosynthetic mechanism (Ehleringer & Björkman, 1977; Sage & Kubien, 2007; Reich et al., 2018). C_4_ species have a C-accumulation strategy, which minimizes photorespiration through biochemical and anatomical specializations. Using this strategy, C_4_ species can concentrate CO_2_ at the active site of the ribulose-1,5-bisphosphate carboxylase-oxygenase (RuBisCO) (Sage, 2004) and be virtually CO_2_ saturated even at the current atmospheric CO_2_ concentration (Tom-Dery et al., 2018). In addition, photosynthetic capacity of many plant species reduced, when they were exposed to elevated CO_2_ due to an inhibition mechanism called photosynthetic acclimation, generally attributed to an alteration in the balance of the supply and sink of assimilates leading to increased nonstructural carbohydrates content in the leaves (Drake, González-Meler & Long, 1997; Porras et al., 2017). However, indirect effects of elevated CO_2_ can increase leaf CO_2_ assimilation rate and growth of C_4_ species via increases in intracellular partial pressure, changes in fixation patterns, improvements of shoot water relations, and increases in leaf temperature (Ghannoum et al., 2000). In addition, recent results from a 20-year free air carbon dioxide enrichment (FACE) experiment showed a much more positive response of C_4_ species to elevated atmospheric CO_2_ concentration compared to C_3_ grasses after 12 years of exposure (Reich et al., 2018).

The response of grasslands to climate change is complex due to the interactions between water availability and management practices (Chang et al., 2017). Higher concentrations of atmospheric CO_2_ have the potential to alter food and fodder nutritional quality (Porteaus et al., 2009; Abdelgawad et al., 2014; Myers et al., 2014; Dumont et al., 2015). Some authors have already noticed that elevated CO_2_ can improve pasture productivity at the expense of a decreased nutritional quality of forage (Milchunas et al., 2005; Mueller et al., 2016; Augustine et al., 2018) and this could result in low production and reproduction rates of grazing livestock since these animals usually depend exclusively on forages to meet their nutritional requirements (Ezzat, Fadlalla & Ahmed, 2018).

Changes in production and chemical composition of plants have significant impact on ecological processes (Damatta et al., 2010; Sanz-Sáez et al., 2012; Abdelgawad et al., 2014). Effects of CO_2_ enrichment on plants have been intensively investigated in C_3_ species (Lara & Andreo, 2011), but some recent studies have investigated these effects on tropical C_4_ grasses (McGranahan & Yurkonis, 2018; Santos et al., 2014; Tom-Dery et al., 2018). These C_4_ grasses are the main fodders in the Brazilian livestock production system, having the largest commercial herd of ruminants with nearly 211 million head (15% of world’s beef production) in an area of approximately 170 million hectares of grasslands (Instituto Brasileiro de Georgrafia e Estatística, 2012; Barneze et al., 2014; Dick, Da Silva & Dewes, 2015; Cerri et al., 2016). Most of this area is under native African C_4_ grass species, mostly belonging to the genus *Brachiaria* (Gracindo et al., 2014). Although the livestock sector in Brazil is one of the largest in the world, little information is available regarding the changes in pasture productivity and forage nutritional quality due to enhanced atmospheric CO_2_.

Therefore, the main objective of this study was to investigate changes in pasture productivity and forage nutritional quality of *Brachiaria decumbens* (cv. Basilisk) in response to 30-month exposure to elevated atmospheric CO_2_. We hypothesized that elevated concentration of atmospheric CO_2_ may result in increased pasture productivity, changes in morphological characteristics and changes in nutritional quality of the forages.

## Materials and Methods

### Study site and FACE facility

**Figure 1:**
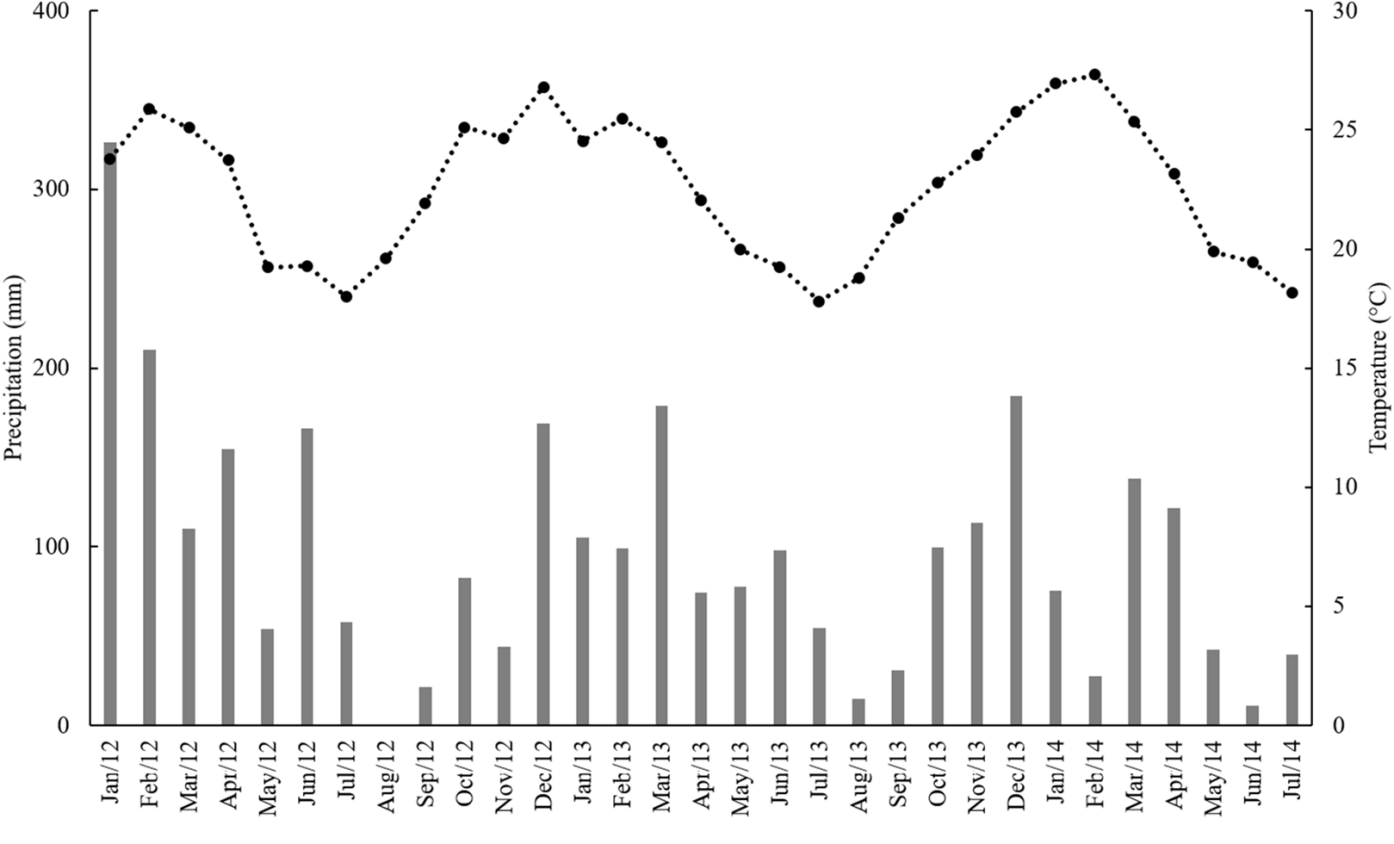
Monthly precipitation (mm) (gray bars) and mean temperature (°C) (black squares) from January 2012 to July 2014.

This experiment was carried out in an experimental area belonging to the Brazilian Agricultural Research Corporation (Embrapa—Meio Ambiente) located in the municipality of Jaguariúna (22°43′S, 47°01′W, 570 m a.s.l), State of São Paulo, Brazil. According to the Köppen classification, the climate is humid subtropical (Cfa), with hot rainy summers and cold dry winters (Fig. 1). The soil is classified as dystrophic Red Latosol with clayey texture, according to the Brazilian soil classification system. Soil physico-chemical properties of the site are described in Maluf et al. (2015).

A total of 12 octagonal rings with 10 m diameter were established in a seven-ha coffee plantation (*Coffea arabica* cv. Catuaí vermelho IAC 144) and forage growth was investigated in a FACE experiment. Six rings were considered as control under normal atmospheric CO_2_ (390 μmol mol^−1^ CO_2_; δ^13^C = −8‰) and the other six rings were under elevated atmospheric CO_2_ (550 μmol mol^−1^ CO_2_; δ^13^C = −10.6‰). The level of atmospheric CO_2_ concentration in elevated atmospheric CO_2_ treatments was increased with an arrangement of tubes and wireless network controlled by environmental sensors (WXT520 climate sensor and GMM343 CO_2_ sensor from Vaisala Co., Helsinki, Finland) through the injection of pure CO_2_ (δ^13^C = −30.7‰) as described by Ghini et al. (2015). CO_2_ enrichment was began in August 2011. The level used in this study (550 μmol mol^−1^ CO_2_) is based on the intermediate scenario (RCP6.0) by 2,070 (International Panel on Climate Change, 2013) and have already affected some crops (McGranahan & Poling, 2018).

### Forage sampling and chemical analysis

Within each ring, two experimental square plots (0.25 m^2^) were cultivated with *B. decumbens* (cv. Basilisk) sown in the last week of October 2011, and after a cut for standardization in January 2012, forage availability was evaluated every 21 days (giving priority to forage nutritional quality) from February 2012 to January 2014 (Abdalla et al., 2016). In October 2012, and October 2013, the experimental plots were fertilized with 40 kg ha^−1^ of N, 82 kg ha^−1^ of P_2_O_5_ and 41 kg ha^−1^ of K_2_O.

A total of 30 months after cultivation under normal and elevated CO_2_ concentrations, average height of grasses was measured with a graduated ruler. All the plants inside the squared plots (0.25 m^2^) were cut at 20 cm above soil surface with scissor.

Biomass production at the field scale was estimated by weighing the collected samples and then they were immediately moved to Animal Nutrition Laboratory (LANA/CENA) for analysis. Samples were then splitted into two portions; the first portion, whole forage was kept intact and the second, the leaf, true stem, inflorescence, and senescence fractions were manually separated. All samples were dried at 55 °C for 72 h. Forage dry matter biomass was weighed, and proportions (%) of leaf, stem, inflorescence, and senescence material were calculated.

For chemical composition analysis, the whole forage and the different fractions were ground in a Wiley mill through a one mm screen. Organic matter (OM) concentrations were determined according to AOAC (2011). Neutral detergent fiber (aNDFom) was analyzed according to Mertens (2002), and acid detergent fiber (ADFom) and lignin (Lignin (sa)) were determined sequentially following the methodology of Van Soest, Robertson & Lewis (1991) using a fiber analyzer (Tecnal - Equipamentos para Laboratórios, Tecnal TE-149, Piracicaba, Brazil) and Ankom filter bags (ANKOM Technology, Ankom F-57, Macedon, NY, USA). Hemicellulose and cellulose were calculated by the differences between aNDFom, ADFom, and Lignin-sa.

To determine the total content of C and N, and N isotopic composition in the forage, samples were ground to pass through a 0.15 mm sieve, sealed in tin capsules and loaded into an elemental analyzer (CH-1110; Carlo Erba, Milan, Italy) for combustion under continuous flow of He. The gases generated from the combustion (CO_2_-C and N_2_-N) were passed directly through the inlet of a mass spectrometer (Thermo Scientific, Delta Plus; Bremen, Germany) and the stable isotopic ratio was expressed using the following equation:

$${\rm\delta}^{15}{\rm{N}}\left( \permil \right) = \left[ {\left( {{{\rm{R}}_{{\rm{sample}}}}/{{\rm{R}}_{{\rm{standard}}}}} \right) - 1} \right] \times {10^3}$$

where R_sample_ and R_standard_ are ^15^N:^14^N ratios of the sample and the standard. Atmospheric N was used as standard for δ^15^N.

Energy dispersive X-ray fluorescence (Shimadzu EDX 720 spectrometer, furnished with a 50 W Rh Anode X-ray tube) technique was used for elemental analysis of potassium (K), calcium (Ca), sulfur (S), phosphorus (P), iron (Fe), and manganese (Mn). The ground samples (*n* = 24) were analyzed under vacuum using a Rh X-ray tube at 50 kV and auto-tunable current adjusted for a detector deadtime below 30% and a collimator with three mm beam size. The X-ray spectrum of the sample was acquired utilizing a Si (Li) detector for 300 s and the quantification was carried out using the fundamental parameters approach.

### Statistical analysis

The experiment was a completely randomized block design (spatial distribution of the rings within the experimental area) with two treatments (Control and Elevated CO_2_) and six replications, and the statistical analysis was performed using SAS software, version 9.4 (SAS Institute Inc., Cary NC, USA). The data were subjected to analysis of variance (ANOVA) using the PROC ANOVA procedure considering block and treatment as fixed effects and the least square means were compared with LSD (*p* < 0.05).

## Results

Elevated CO_2_ had no effect on biomass productivity, average height, and proportions of leaf, stem, senescence, and inflorescence (Table 1). The OM and fiber fractions of the whole forage, leaf, stem, and senescence portions were also not influenced by elevated CO_2_ concentration (Table 2). Elevated CO_2_ showed a nonsignificant (*p* > 0.05) 18% decrease in biomass productivity, 10% decrease in average height, 4% decrease in FDAom and 5% decrease in Lignin (sa) related to control.

**Table 1:** Biomass productivity, average height, leaf, stem, senescence, and inflorescence proportions of *Brachiaria decumbens* cv. Basilisk grown under contrasting atmospheric CO_2_ concentrations.

Parameters	Treatments		95% CI	*p*-value
	Control (*n* = 24)	Elevated CO_2_ (*n* = 24)		
Biomass productivity (kg FM ha^−1^)	28,657.6 ± 4,008.26	26,765.4 ± 3,554.44	[−14856–11072]	0.7619
Biomass productivity (kg DM ha^−1^)	5,654.5 ± 592.63	4,609.5 ± 610.67	[−2811.18–721.05]	0.2288
Average height (cm)	60.2 ± 2.41	53.7 ± 3.84	[−16.53–3.65]	0.1960
Leaf proportion (%)	38.5 ± 2.31	39.6 ± 2.21	[−5.75–7.87]	0.7471
Stem proportion (%)	48.5 ± 3.36	44.7 ± 1.65	[−11.94–4.19]	0.3249
Senescence proportion (%)	20.2 ± 1.89	21.1 ± 2.19	[−5.17–6.93]	0.7620
Inflorescence proportion (%)	3.0 ± 0.58	3.8 ± 1.06	[−1.58–3.16]	0.4915

**Note:**

Means ± standard error of the means; FM, fresh matter; DM, dry matter; Treatments: control, ambient conditions (≈390 μmol mol^−1^ CO_2_), elevated CO_2_, CO_2_ fertilization (≈550 μmol mol^−1^ CO_2_); CI, confidence interval.

**Table 2:** Organic matter and fiber fractions of *Brachiaria decumbens* cv. Basilisk grown under contrasting atmospheric CO_2_ concentrations.

Parameters (g kg^−1^ DM)	Treatments		95% CI	*p*-value
	Control (*n* = 24)	Elevated CO_2_ (*n* = 24)		
Whole plant				
OM	945.1 ± 1.70	944.4 ± 1.74	[−5.49–4.20]	0.7835
aNDFom	682.6 ± 8.02	671.9 ± 8.57	[−35.88–14.41]	0.3836
ADFom	398.5 ± 10.32	382.9 ± 8.80	[−42.17–11.02]	0.2983
Lignin (sa)	81.5 ± 3.20	77.4 ± 3.08	[−12.64–4.62]	0.3413
HEMI	284.1 ± 6.07	289.0 ± 3.36	[−10.38–20.16]	0.5083
CEL	317.0 ± 8.78	305.5 ± 6.77	[−35.00–11.92]	0.3139
Leaf portion				
OM	939.5 ± 1.41	942.3 ± 1.07	[−0.69–6.33]	0.1090
aNDFom	555.1 ± 7.00	564.1 ± 6.55	[−7.36–25.38]	0.2619
ADFom	274.9 ± 3.92	279.8 ± 5.13	[−8.10–17.85]	0.4389
Lignin (sa)	64.0 ± 4.99	67.6 ± 5.18	[−8.36–15.40]	0.5407
HEMI	280.2 ± 4.28	284.3 ± 5.43	[−9.25–17.53]	0.5230
CEL	210.9 ± 5.05	212.2 ± 6.26	[−15.74–18.46]	0.8689
Stem portion				
OM	957.4 ± 1.71	957.9 ± 1.50	[−3.46–4.44]	0.7964
aNDFom	778.0 ± 5.70	773.6 ± 7.40	[−22.22–13,39]	0.6075
ADFom	495.7 ± 6.16	491.7 ± 8.68	[−27.35–19.27]	0.7190
Lignin (sa)	107.4 ± 5.64	118.7 ± 17.41	[−28.50–51.13]	0.5567
HEMI	282.3 ± 4.07	281.9 ± 3.13	[−12.32–11.52]	0.9444
CEL	388.3 ± 5.91	373.0 ± 15.56	[−48.97–18.26]	0.3486
Senescence portion				
OM	931.1 ± 2.05	926.6 ± 4.07	[−12.52–9.67]	0.7896
aNDFom	739.8 ± 4.31	730.1 ± 5.26	[−23.43–11.12]	0.4612
ADFom	458.9 ± 9.16	445.4 ± 6.47	[−30.95–16.00]	0.5094
Lignin (sa)	91.0 ± 4.35	94.9 ± 3.54	[−7.44–13.45]	0.5502
HEMI	280.9 ± 5.71	284.6 ± 4.93	[−13.34–16.03]	0.8487
CEL	367.8 ± 5.89	350.5 ± 4.98	[−29.60–8.65]	0.2629

**Note:**

Means ± standard error of the means; DM, dry matter; OM, organic matter; aNDFom, neutral detergent fiber; ADFom, acid detergent fiber; Lignin (sa), Lignin; HEMI, hemicellulose; CEL, cellulose; Treatments: control, ambient conditions (≈390 μmol mol^−1^ CO_2_), elevated CO_2_, CO_2_ fertilization (≈550 μmol mol^−1^ CO_2_); CI, confidence interval.

Despite no significant effect on C and N concentrations, a decrease (*p* < 0.05) in the C:N ratio of the whole plant was observed under elevated CO_2_ (Table 3). In addition, elevated CO_2_ led to higher values of δ^15^N in the leaf and stem portions of *B. decumbens* (*p* < 0.05). However, such increase (0.4‰ and 0.7‰ in leaf and stem portions, respectively) was generally lower or very close to 0.5‰, which is the analytical error of this analysis.

**Table 3:** Multielemental and isotopic composition of *Brachiaria decumbens* cv. Basilisk grown under contrasting atmospheric CO_2_ concentrations.

Parameters	Treatments		95% CI	*p*-value
	Control (*n* = 24)	Elevated CO_2_ (*n* = 24)		
Whole plant				
C (%)	42.95 ± 0.228	42.22 ± 0.447	[−1.78–0.32]	0.1624
N (%)	1.9 ± 0.072	2.12 ± 0.094	[−0.03–0.48]	0.0885
C:N	22.97 ± 0.8^a^	20.36 ± 0.896^b^	[−5.17−0.04]	0.0462
δ^15^N (‰)	4.4 ± 0.107	4.4 ± 0.152	[−0.42–0.43]	0.9680
K (%)	1.43 ± 0.11	1.57 ± 0.066	[−0.14–0.42]	0.3308
Leaf portion				
C (%)	43.49 ± 0.221	43.76 ± 0.149	[−0.31–0.85]	0.3397
N (%)	2.78 ± 0.074	2.84 ± 0.059	[−0.14–0.27]	0.5278
C:N	15.82 ± 0.49	15.46 ± 0.318	[−1.62–0.96]	0.5776
δ^15^N (‰)	4.2 ± 0.104^b^	4.6 ± 0.118^a^	[0.08–0.81]	0.0188
K (%)	1.88 ± 0.071	1.83 ± 0.054	[−0.24–0.13]	0.5363
Stem portion				
C (%)	41.35 ± 0.181	41.57 ± 0.142	[−0.18–0.61]	0.2782
N (%)	1.43 ± 0.051	1.43 ± 0.063	[−0.17–0.16]	0.9898
C:N	29.32 ± 1.097	29.76 ± 1.359	[−3.19–4.08]	0.7998
δ^15^N (‰)	4.2 ± 0.16^b^	4.9 ± 0.138^a^	[0.13–1.11]	0.0162
K (%)	1.66 ± 0.086	1.72 ± 0.080	[−0.20–0.32]	0.6161
Senescence portion				
C (%)	40.8 ± 0.175	40.5 ± 0.36	[−1.23–0.63]	0.5006
N (%)	1.24 ± 0.048	1.32 ± 0.048	[−0.05–0.22]	0.2386
C:N	33.15 ± 1.279	31.07 ± 1.23	[−5.69–1.13]	0.1755
δ^15^N (‰)	4.6 ± 0.151	5.1 ± 0.163	[−0.10–0.97]	0.1053
K (%)	1.2 ± 0.056	1.29 ± 0.069	[−0.11–0.30]	0.3626

**Note:**

Means ± standard error of the means; ^a,b^Different letters in the same row indicate statistical difference; Treatments: control, ambient conditions (≈390 μmol mol^−1^ CO_2_); Elevated CO_2_, CO_2_ fertilization (≈550 μmol mol^−1^ CO_2_); CI, confidence interval.

**Figure 2:**
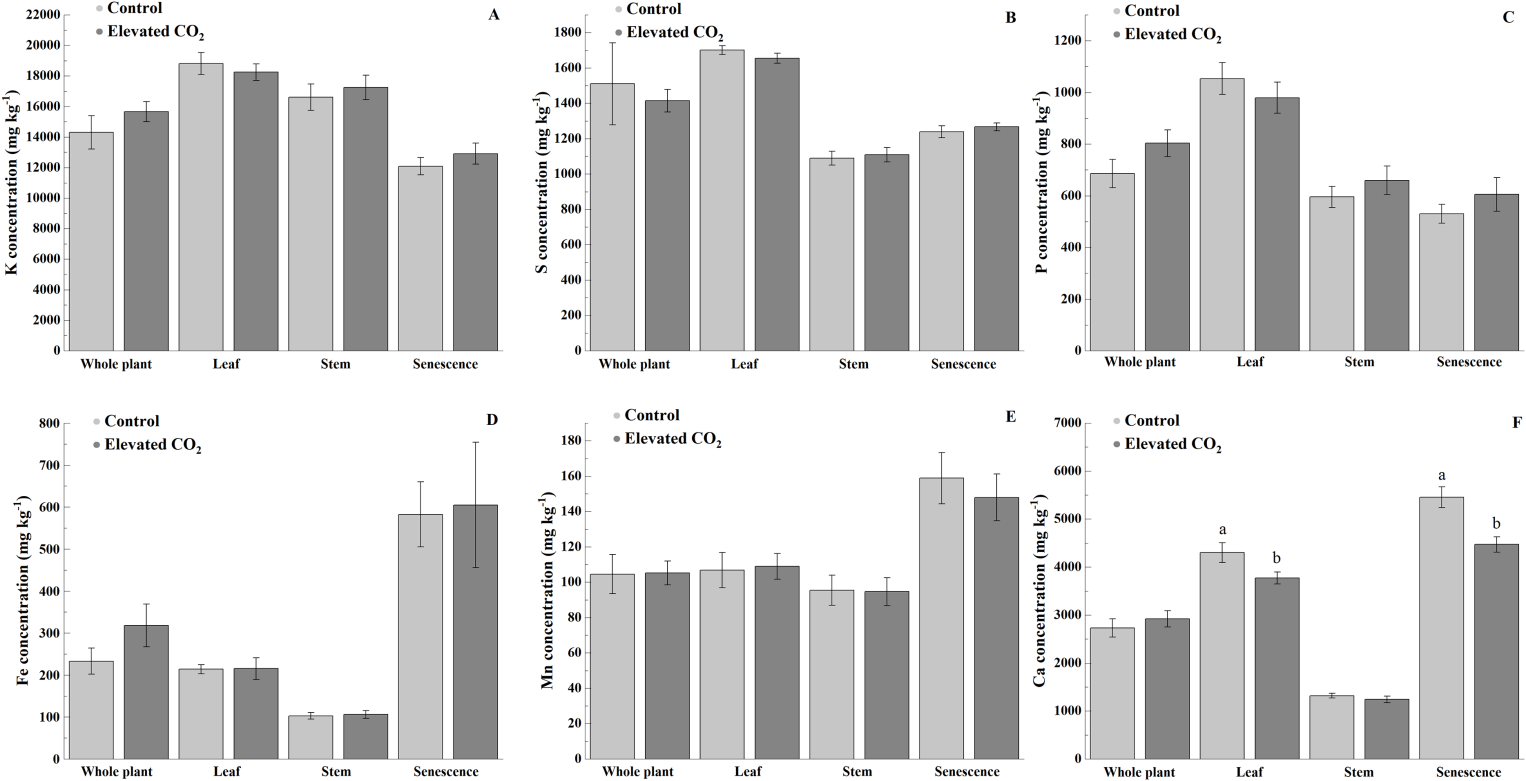
Concentrations (mg kg^−1^) of K (A), S (B), P (C), Fe (D), Mn (E), and Ca (F) on the whole plant, leaf, stem, and senescence portions of *Brachiaria decumbens* cv. Basilisk grown under contrasting CO_2_. Control—Ambient conditions (≈390 μmol mol^−1^ CO_2_), Elevated CO_2_—CO_2_ fertilization (≈550 μmol mol^−1^ CO_2_). Bars are standard error of the means (*n* = 12).

Elevated CO_2_ did not influence the concentrations of K, S, P, Fe, and Mn in the whole plant, leaf, stem, and senescent portions (Figs. 2A–2E). However, lower concentration of Ca was observed in the leaf (12%) and senescence portion (18%) of *B. decumbens* grown under elevated atmospheric CO_2_ (*p* < 0.05) (Fig. 2F).

## Discussion

Even after two and half years of exposure to elevated atmospheric CO_2_ concentration, the productivity, and morphological characteristics of *B. decumbens* (cv. Basilisk) were not influenced, following the same pattern observed in our trial with *B. brizantha* (cv. Marandu) in a short term (9 months) experiment (A. L. Abdalla Filho, P. M. T. Lima, G. Z. Sakita, T. P. D. Silva, W. S. Costa, R. Ghini, A. L. Abdalla, M. C. Piccolo, 2019, unpublished data). These results rejects our initial hypothesis and are in line with Dumont et al. (2015) stating that only N content and nonstructural carbohydrates concentrations increased under elevated CO_2_ in a meta-analysis. No effects of elevated CO_2_ (950 μmol mol^−1^ CO_2_) on total biomass of the C_4_ grass *Cenchrus pedicellatus* were also observed by Tom-Dery et al. (2018). However, a recent study conducted over 20 years in a FACE facility in Minnesota, USA, showed that after 12 years of exposure to elevated CO_2_, the total biomass of several C_4_ grasses enhanced (Reich et al., 2018). Effects of elevated CO_2_ could also vary according to the evaluated cultivar. Using growth chambers and the same concentration level of our study (550 μmol mol^−1^ CO_2_), Santos et al. (2014) evaluated three cultivars of buffel grass (*Cenchrus ciliaris*) and found that elevated CO_2_ did not affect the productive characteristics of Biloela, decreased Aridus forage mass and increased forage mass in West Australia. The reason for discrepancies between different studies is unknown but it is important to consider the differences in methodologies as a possible explanation (McGranahan & Yurkonis, 2018) since growth chambers may overestimate the effects of elevated CO_2_ on photosynthetic and plant growth parameters (Leakey et al., 2006).

Plants respond directly to higher atmospheric CO_2_ concentration through photosynthesis and stomatal conductance, and these are the basis for the higher biomass production (Long et al., 2006). However, in C_4_ plants, RuBisCO is localized in bundle sheath cells, in which CO_2_ is concentrated in levels of three to six times higher than those of the atmospheric CO_2_ concentration (Caemmerer & Furbank, 2003). Such CO_2_ enrichment is sufficient to saturate RuBisCO and prevent any increase in CO_2_ uptake with CO_2_ fertilization. In addition, under elevated CO_2_ condition, C_4_ plants can close their stomata to reduce water loss during photosynthesis (McGranahan & Poling, 2018). Still, sufficient rainfall during the experimental period (Fig. 1) could limit gains from reduced transpiration (Fay et al., 2012). For these reasons and the fact that we used a FACE facility, we observed the lack of response to elevated CO_2_ in our study.

Structural carbohydrates in *B. decumbens* were also not affected by the increased atmospheric CO_2_ concentration, refuting our hypothesis. These results are in line with Dumont et al. (2015) but contradicts the findings of Abdalla et al. (2016) evaluating this cultivar under the same treatments (control and elevated CO_2_) during the rainy season. Our results also contradict Tom-Dery et al. (2018) stating that elevated CO_2_ reduced Lignin and increased ADF content of *Cenchrus pedicellatus*. It is noteworthy that in our study, in order to evaluate the effect of 30 month exposure to elevated atmospheric CO_2_, the grass was kept under no grazing management (e.g., considering the concept of critical leaf area index to determine the time of sampling) for almost 6 months, which resulted in older plants with higher proportion of stem, as well as fiber fractions compared to the other studies evaluating the same cultivar (Pedreira, Braga & Portela, 2017; Lima et al., 2018).

By altering plant and microbial processes involved in the N cycle, elevated atmospheric CO_2_ may change the isotopic signature of plant N (Polley et al., 2015). In our study, a slightly lower C:N ratio was found when the whole plant was analyzed (Table 3). Similarly, rather higher δ^15^N values were found under elevated CO_2_ in some plant parts. In another study, higher δ^15^N in leaves of ponderosa pine with increasing atmospheric CO_2_ was recorded (Johnson, Cheng & Ball, 2000); meanwhile, in our study the difference was significant but it was too small to be attributed to elevated CO_2_.

The major difference was a reduced Ca content of the leaves and the senescence portion of *B. decumbens* under elevated atmospheric CO_2_. Calcium is an essential macronutrient for plant growth, plays an important structural role in the cell wall and membranes, and acts as an intracellular messenger in the cytosol (White & Broadley, 2003). Lower levels of Ca in forage may have implications for animal nutrition since reduced availability of Ca to the rumen microbes decreases fiber digestion (Fielding & Miller, 1986). The Ca content of forages is the net result of absorption and translocation processes operating within the roots and shoots and such processes are being modulated by various environmental factors affecting plant growth and metabolism (Grunes & Welch, 1989).

Other studies found lower Ca concentration in sorghum and soybean under elevated atmospheric CO_2_ due to the dilution effect caused by an increased biomass (Rogers, Runion & Krupa, 1994; Rogers et al., 1999), often referred as the “dilution hypothesis” (Loladze, 2014). As the yield was not changed due to the elevated CO_2_ in our study, other mechanisms may be involved in reduced Ca concentration. Related to the flow of nutrients, the processes involved in the use of available water may be affected by elevated CO_2_ concentration since under this condition, transpiration rates of plants may be reduced and water use efficiency in photosynthetic processes may be improved (Fay et al., 2012). In this study, the plots were kept under similar soil fertility, daily air temperature and rainfall conditions, and the only different parameter was the concentration of atmospheric CO_2_, hence the reduced Ca content under elevated CO_2_ is more related to an enhanced water use efficiency (parameter not evaluated here) rather than the dilution hypothesis. Despite the reduced Ca content of *B. decumbens* under elevated CO_2_, it is important to emphasize that a possible Ca deficiency in ruminants can be easily ameliorated by feeding calcium-containing mineral supplements (e.g., limestone, steamed bone flour, and dicalcium phosphate) (McDonald et al., 2011).

The predicted world population of 9.6 billion in the next decades will result in 70% increase in the demand of animal derived foods and considering the current scenario of climate change, sustainable production of them to achieve food security will be a big challenge faced by humanity (Gerber et al., 2013; Cerri et al., 2016). Our results showed that the productivity and fiber fractions of *B. decumbens* were not impaired by elevated CO_2_, suggesting that the tropical pasture-based beef production has the potential to overcome the above-mentioned challenges. A remarkable sustainable potential of grazing systems is also shown in recent studies (De Oliveira Silva et al., 2016; Dass et al., 2018).

## Conclusions

We concluded that productivity, morphological characteristics, and fiber fractions of *B. decumbens* (cv. Basilisk) were not affected by elevated atmospheric CO_2_ in 30 months. These results are of great importance since *B. decumbens* is one of the main fodders in the Brazilian livestock production system, where extensive grazing is predominant and the herds depend almost exclusively on these grasses to meet their nutritional requirements. However, elevated CO_2_ decreased forage Ca content, which can affect livestock production under a climate change scenario and needs further investigations.

## Supplemental Information

10.7717/peerj.5932/supp-1 Supplemental Information 1 Raw data.

## Additional Information and Declarations

### Data Availability

The following information was supplied regarding data availability: The raw data are provided in a Supplemental File.
